# RHOJ Induces Epithelial-to-Mesenchymal Transition by IL-6/STAT3 to Promote Invasion and Metastasis in Gastric Cancer

**DOI:** 10.7150/ijbs.81972

**Published:** 2023-08-21

**Authors:** Zhijie Ma, Qi Sun, Chengfei Zhang, Qian Zheng, Yixuan Liu, Haojun Xu, Yiting He, Chengyun Yao, Jinfei Chen, Hongping Xia

**Affiliations:** 1 Zhongda Hospital, School of Medicine & Advanced Institute for Life and Health, Southeast University, Nanjing 210009, China.; 2 Department of Pathology, Nanjing Drum Tower Hospital Clinical College of Nanjing Medical University, Nanjing 210008, China.; 3 School of Basic Medical Sciences & Key Laboratory of Antibody Technique of National Health Commission & Jiangsu Antibody Drug Engineering Research Center, Nanjing Medical University, Nanjing 211166, China.; 4 Sir Run Run Hospital, Nanjing Medical University, Nanjing 211166, China.; 5 Jiangsu Cancer Hospital, The Affiliated Cancer Hospital of Nanjing Medical University, Jiangsu Institute of Cancer Research, Nanjing 210009, China.; 6 Department of Oncology, the First Affiliated Hospital of Wenzhou Medical University, Wenzhou, 325015, Zhejiang, China.

**Keywords:** Gastric Cancer, RHOJ, EMT, IL-6/STAT3 signaling, Metastasis

## Abstract

**Background:** Recently, the molecular classification of gastric cancer (GC) promotes the advances of GC patients' precision therapy and prognosis prediction. According to the Asian Cancer Research Group (ACRG), GC is classified as microsatellite instable (MSI) subtype GC, microsatellite stable/epithelial-to-mesenchymal transition (MSS/EMT) subtype GC, MSS/TP53- subtype GC, and MSS/TP53+ subtype GC. Due to the easy metastasis of EMT-subtype GC, it has the worst prognosis, the highest recurrence rate, and the tendency to occur at a younger age. Therefore, it is curious and crucial for us to understand the molecular basis of EMT-subtype GC.

**Methods:** The expression of RHOJ was detected by quantitative real-time PCR (qPCR) and immunohistochemistry (IHC) in GC cells and tissues. Western blotting and immunofluorescence (IF) were conducted to examine the effects of RHOJ on the EMT markers' expression of GC cells. The GC cells' migration and invasion were investigated by transwell assay. The tumor growth and metastasis were demonstrated correspondingly in different xenograft models.

**Results:** Firstly, it was noticed that RHOJ was significantly upregulated in EMT-subtype GC and RHOJ has close relationships with the EMT process of GC, based on the Gene Expression Omnibus (GEO) and the Cancer Genome Atlas (TCGA) databases. Next, transwell assay and tail vein metastasis models were conducted to verify that RHOJ mediates the EMT to regulate the invasion and metastasis of GC *in vitro* and *in vivo*. In addition, weakened tumor angiogenesis was observed after RHOJ knockdown by the angiogenesis assay of HUVEC. RNA-seq and further study unveiled that RHOJ aggravates the malignant progression of GC by inducing EMT through IL-6/STAT3 to promote invasion and metastasis. Finally, blocking the IL-6/STAT3 signaling overcame RHOJ-mediated GC cells' growth and migration.

**Conclusions:** These results indicate that the upregulation of RHOJ contributes to EMT-subtype GC invasion and metastasis via IL-6/STAT3 signaling, and RHOJ is expected to become a promising biomarker and therapeutic target for EMT-subtype GC patients.

## Introduction

Remaining one of the most common malignant tumors worldwide, Gastric Cancer (GC) ranks fifth for incidence and fourth for mortality globally 1. Due to its delayed diagnosis, highly aggressive and metastatic ability, and anti-chemotherapy, the 5-year survival rate of GC patients is only about 30% 2, 3. Thus, it is urgent to understand the molecular pathogenesis deeply and explore more effective targeted therapies of GC. Currently, most GC patients' treatments are terrible because patients are commonly treated in a uniform dosage regimen irrespective of disease subtype. While the traditional histopathologic classification of GC can sometimes influence the options of treatment schemes, they remain insufficient to guide precise treatments for individual patients 4. Fortunately, the development of molecular pathology has gradually contributed to the clinical diagnosis and treatment of GC. The Asian Cancer Research Group (ACRG) newly proposed a classification system where GC is divided into four subtypes: microsatellite instable (MSI) subtype, microsatellite stable/epithelial-to-mesenchymal transition (MSS/EMT) subtype, microsatellite stable/the tumor protein 53 active (MSS/TP53+) subtype, and microsatellite stable/the tumor protein 53 inactive (MSS/TP53-) subtype 5, 6.

Characterized by the high cellular motility, the EMT-subtype GC owns loss of CDH1 expression and the upregulation expression of classic genes that correlated with mesenchymal cell phenotype. EMT is a complex biological process in which epithelial cells acquire mesenchymal characteristics, including loss of cellular adhesion junctions and substrate polarity; the appearance of spindle-shaped cell morphology and up expression of the related mesenchymal protein (Vimentin, N-cadherin, etc); reinforcement of cellular motility and invasion ability; and resistance to cell apoptosis 7, 8. This transition usually occurs during embryonic development and organ differentiation, but can also observe in pathological states, such as chronic inflammation, maintenance of tumor stemness, tumorigenesis, and metastasis. In cancer, EMT is considered an essential contributor to tumor initiation, invasion, metastasis, and resistance to therapy 9-11. Moreover, a study reported that EMT endows tumorigenicity to mice breast cancer cells through the upregulated expression of vascular endothelial growth factor A (VEGFA) and through enhancing tumor angiogenesis effect 12. Due to the enhancement of tumor cellular motility, the EMT-subtype GC has the worst prognosis, the highest recurrence rate (approximately 63%) of the four subtypes, and the tendency to occur at a younger age 6, 13. Therefore, it is curious and essential for us to understand the molecular basis of EMT-subtype GC.

In this study, we identified that RHOJ is upregulated aberrantly in EMT-subtype GC and was negatively correlated with GC patient prognosis. Highly enriched in endothelial cells (ECs), RHOJ is one of the members of the RHO guanosine triphosphatases (RHO GTPases) family 14, 15. As a cell membrane protein, like a molecular switch between active GTP-bound and inactive GDP-bound forms, RHO GTPases regulate a series of cellular processes related to their key roles in controlling the cytoskeleton, such as cell morphology and polarity, cell motility, vesicle trafficking, and cytokinesis 16-19. Relevant studies have shown RHO GTPases signaling contributes to the malignant progression of cancer and some RHO GTPases emerge as a potential therapeutic target 20-23, and a new study has uncovered that RHOJ acts as a key regulator of EMT-associated resistance to chemotherapy 24. In addition, RHOJ interruption has been reported as an effective therapeutic option for targeting the tumor vascular system 25-27. Our recent studies also showed that RHOJ is significantly overexpressed in glioblastoma and facilitates angiogenesis and tumor invasion 28, 29. Here, we first investigated that RHOJ mediates the EMT to regulate the invasion and metastasis of GC *in vitro* and *in vivo*. Meanwhile, the promoting effects of RHOJ in GC angiogenesis and proliferation have been tested as well. Further studies revealed that RHOJ promotes EMT-mediated invasion and metastasis of GC via the IL-6/STAT3 signaling, and showed a possibility that RHOJ is a selective prognostic biomarker and a promising therapeutic target for GC patients.

## Materials and methods

### Transwell assay

Cell migration and invasion assay were conducted by transwell chambers (TCS013024, Biofil, Guangzhou, China) with a filter of 8 μm pore and matrigel (40183ES08, Yeasen, Shanghai, China). For migration assay, after being cultured with serum-free DMEM medium for 6 h, 5×10^4^ cells with 200 μL serum-free DMEM medium were seeded in the upper chamber of a 24-well plate and 600 μL DMEM medium containing 20% FBS was added to the lower chamber. After incubating for appropriate moments, migrated cells were fixed with 4% paraformaldehyde for 40 min and stained with 0.1% crystal violet for 40 min. Then washing with water and using cotton swabs to erase cells that stayed in the upper chamber, migrated cells were randomly captured under a microscope and counted by Image J software. For invasion assay, approximately 1 h before the experiment began, 40 μL diluted Matrigel was dripped in the upper chamber and placed at 37°C for solidification, and the rest steps were the same as above.

### Cell cycle analysis

Cells were harvested at 2 days after planting in 6-well plates (1.5×10^5^ cells/well), washed twice with PBS, and fixed overnight with 70% ethanol (PBS diluted) at -20°C. After being stained with propidium iodide (PI), cells were analyzed by a flow cytometer (FACSVerse, BD, USA) from the Analysis and Testing Center at Nanjing Medical University (NMU).

### Cell apoptosis analysis

Supernatant DMEM medium and cells were collected, then cell apoptosis was detected by Annexin V-FITC/PI Apoptosis Detection Kit (40302ES60, Yeasen, Shanghai, China) according to the product brochure. Within 1 h, the cell suspension was analyzed using a flow cytometer (FACSVerse, BD, USA) of the Analysis and Testing Center at NMU.

### 5-ethynyl-2'-deoxyuridine (EdU) proliferation assay

The EdU assay was conducted by EdU proliferation testing assay kit (C0075S, Beyotime, Shanghai, China) and DAPI. Cells were seeded in co-focal dishes (2×10^5^ cells/well) and cultured for 24 h, then incubated with EdU for 3 h, and the remaining steps were based on its reagent instructions. After being stained with DAPI, images were captured using fluorescence microscopy.

### Terminal deoxynucleotidyl transferase dUTP nick end labeling (TUNEL) assay

The TUNEL assay was performed by TUNEL Apoptosis Detection Kit (Alexa Fluor 488) (40307ES60, Yeasen, Shanghai, China) and DAPI. Planted in co-focal dishes (2×10^5^ cells/dish) 24 h in advance, then cells were fixed with 4% paraformaldehyde and treated with 150 μL Proteinase K (20 μg/mL) for 5 min. After washing twice with PBS, the remaining procedures were followed with the manufacturers' instructions. Fluorescence microscopy was used to capture the images of TUNEL-positive cells.

### Enzyme-linked immunosorbent assay (ELISA)

ELISA was performed by ELISA MAX™ Standard Set Human IL-6 Detection Kit (430501, Biolegend, USA). The Supernatant of RHOJ overexpression cells (SNU-1) and RHOJ knockdown cells (SNU-1) was collected in advance and stored at -80°C. 16 h before running the ELISA, 100 μL diluted (diluted with Coating Buffer) Capture Antibody was added to 96-well plates, sealed plates, and incubated overnight at 4°C. The subsequent assay procedures were based on its' brochures.

### Animal assay

NCG mice (6 weeks old, male) were obtained from the Model Animal Research Center of NMU and housed in a specific pathogen-free environment. For the xenograft model, NCG mice were classified into four groups randomly: SGC7901-shNC group, SGC7901-shRHOJ group, SNU-1-shNC group, and SNU-1-shRHOJ group (n=7, 7, 5, 5). SGC7901-shNC group (5×10^6^ cells), SGC7901-shRHOJ group (5×10^6^ cells), SNU-1-shNC group (4×10^6^ cells), and SNU-1-shRHOJ group (4×10^6^ cells) were suspended in 200 μL PBS and were injected subcutaneously into the left and right back of per mice. The tumor volume was recorded by Vernier calipers per week (the formula V (mm^3^) = length×width^2^/2). After 6 weeks, tumors were extracted from per mice's back, measured weight, and fixed with 4% paraformaldehyde at 4°C for hematoxylin & eosin (HE) staining, IHC, etc. For the tail vein metastasis model, the grouping of NCG mice (n=7, 7, 6, 7) was the same as above. Cells of each group (1.5×10^6^ cells) were suspended in 200 μL PBS and were injected into the tail vein of per mouse. After 7 weeks, the lung and liver tissues of mice were dissected and photographed, and the number of metastatic lung and liver foci was recorded. Similarly, the mice's lung and liver tissues were fixed in 4% paraformaldehyde at 4°C for further analysis.

### RNA-sequence (RNA-seq)

The global gene expression profiles of RHOJ overexpression and control SNU-1 cells were examined by RNA-seq in Novogene (Beijing, China), and then the Enrichr tool was used to analyze the biological process.

### Statistical analysis

All data were analyzed using the Student's *t*-test of GraphPad Prism 6 and expressed as Mean±SD. Differences were considered to be statistically significant for *P*<0.05. *P*<0.05, *P*<0.01, *P*<0.001 and *P*<0.0001 are denoted by *, **, *** and **** respectively.

## Results

### Upregulated in EMT-subtype GC, RHOJ is correlated with poor GC prognosis

**Figure 1:**
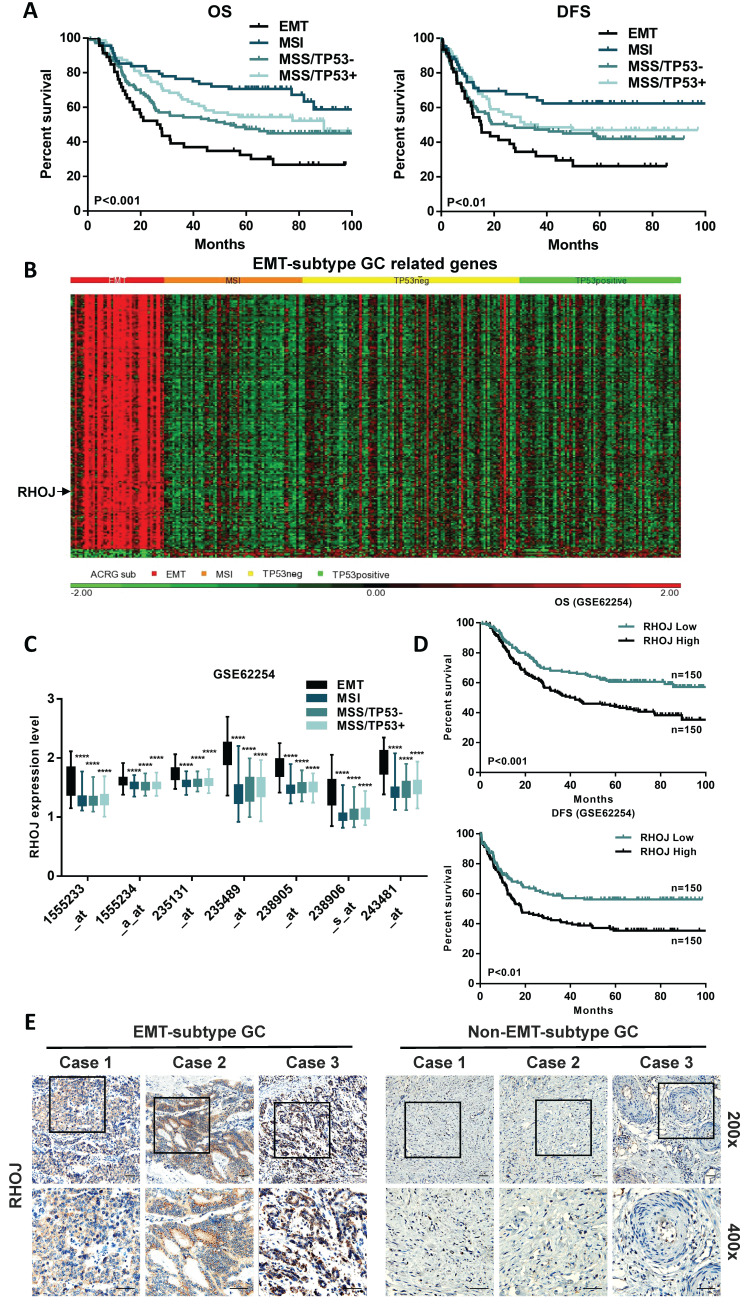
** Upregulated in EMT-subtype GC, RHOJ is correlated with poor GC prognosis.** (A) Kaplan-Meier analysis showed the patients' overall survival (OS) and disease-free survival (DFS) in the four GC subtypes of ACRG, according to the GSE62254 dataset. (B) Heatmap of different expression patterns of EMT-subtype GC-related genes in the four GC subtypes of ACRG. (C) In the GSE62254 dataset, RHOJ (labeled by seven independent molecular probes) expression levels in the four GC subtypes of ACRG. (D) Kaplan-Meier analysis showed the OS and DFS in RHOJ high expression group and low expression group GC patients, according to the GSE62254 dataset. (E) IHC staining tested RHOJ expression levels of the EMT-subtype GC and Non-EMT-subtype GC tissue specimens, scale bar, 50 µm. *P<0.05, **P<0.01, ***P<0.001, ****P<0.0001. Data were expressed as Mean±SD.

According to correlated reports on molecular classifications of GC from the ACRG, we focused on the EMT-subtype GC, which owns the worst prognosis of the four subtypes 6, 13. In the initial study, a Kaplan-Meier analysis of 300 GC patients in the GSE62254 dataset observed that the patients' survival of EMT-subtype GC was shortest both in overall survival (OS) and disease-free survival (DFS) (Figure 1A). To further explore the critical genes that regulate the malignant progression of EMT-subtype GC, the differential genes of the four ACRG subtypes were detected and analyzed (Figure 1B).

Interestingly, compared to the other three subtypes, the RHOJ expression level was significantly upregulated in EMT-subtype GC (Figure 1C and Figure S1A). Meanwhile, Kaplan-Meier analysis showed that GC patients in the high RHOJ expression group own shorter OS and DFS than those in the low RHOJ expression group, according to the GSE62254 dataset (Figure 1D) and the TCGA database (Figure S1B). The ingenuity pathway analysis (IPA) of EMT-subtype GC-related genes also identified the importance of RHO Family GTPases (Figure S1C). As is shown in Figure 1E, the expression level of RHOJ significantly increased in EMT-subtype GC clinical tissues than in Non-EMT-subtype GC clinical tissues. Additionally, the GSE62254 dataset was used to analyze the correlation between the GC clinicopathological features and expression of RHOJ, and results (Table S1) displayed that high RHOJ expression led to a younger age of onset, later TNM stage, easier recurrence, and higher perineural invasion rate of GC. The above results suggest that RHOJ may play a critical role in regulating the malignant progression of EMT-subtype GC patients and RHOJ is expected to become a new prognostic biomarker for GC patients.

### RHOJ mediates EMT to regulate the migration and invasion of GC cells

**Figure 2:**
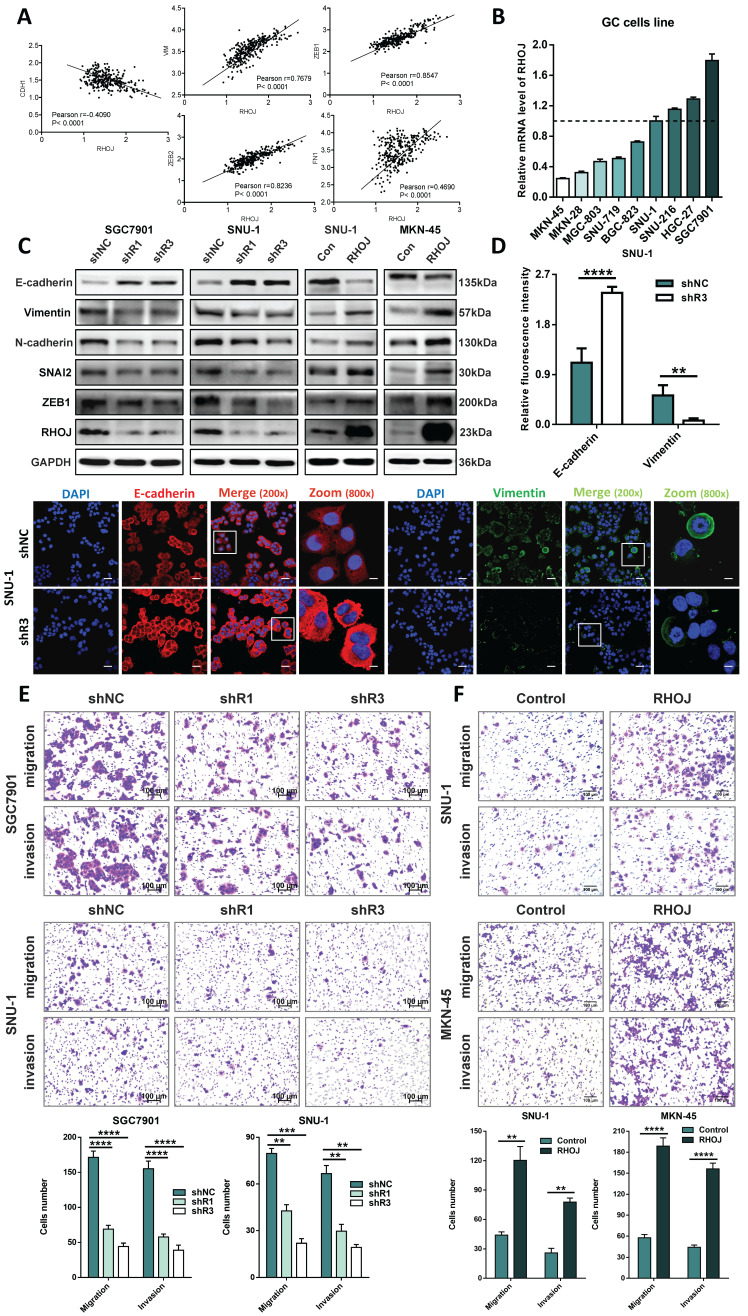
** RHOJ mediates EMT to regulate the migration and invasion of GC cells.** (A) Pearson correlation analysis assessed the links between the expression levels of the EMT-related genes (CDH1, VIM, ZEB1, ZEB2, and FN1) with RHOJ in the GSE62254 dataset. (B) RHOJ relative expression levels of GC cells line were assessed by qPCR. (C) Protein levels of RHOJ, ZEB1, SNAI2, N-cadherin, Vimentin, and E-cadherin were detected by western blotting in RHOJ knockdown cells (SGC7901, SNU-1) and RHOJ overexpression cells (SNU-1, MKN-45). (D) IF staining representative images and quantified charts of E-cadherin (Red), Vimentin (Green), and DAPI (Blue) in SNU-1 cells captured by a confocal microscope, magnification, 200× and 800×, scale bar, 40 µm. (E) Representative images and statistical charts of transwell migration or invasion assay in RHOJ knockdown cells (SGC7901, SNU-1), scale bar, 100 µm. (F) Representative images and statistical charts of transwell migration or invasion assay in RHOJ overexpression cells (SNU-1, MKN-45), scale bar, 100 µm. *P<0.05, **P<0.01, ***P<0.001, ****P<0.0001. Data were expressed as Mean±SD.

Above findings suggest that RHOJ may be involved in the malignant progression of EMT-subtype GC. Given the characteristics of EMT-subtype GC, i.e., loss of CDH1 expression in tumor cells and decreased intercellular adhesion, the high linear correlations between the expression levels of the EMT-related genes (CDH1, VIM, ZEB1, ZEB2, and FN1) with RHOJ both in the GSE62254 dataset (Figure 2A) and the TCGA database (Figure S2A) were observed by Pearson correlation analysis. To clarify this, the RHOJ expression level of the GC cell line was measured by qPCR first (Figure 2B). On this basis, we established stable RHOJ knockdown and RHOJ overexpression GC cells line (SGC7901, SNU-1, and MKN-45) by infection with lentiviral vectors respectively (Figure 2C and Figure S2B). (Initially, three shRNAs were constructed, of which shRHOJ-2 could not effectively inhibit the expression of RHOJ after transfection, and subsequent experiments were merely carried out with shRHOJ-1 and shRHOJ-3). Then the expression of EMT-related proteins was detected by western blotting and IF staining for further verification, and western blotting results demonstrated that RHOJ knockdown by shRNA in SGC7901, SNU-1 cells significantly enhanced the protein level of E-cadherin and decreased the protein levels of Vimentin, N-cadherin, SNAI2, and ZEB1, that were considered classical EMT markers. In contrast, these EMT markers' protein levels showed an opposite trend in RHOJ overexpression SNU-1 and MKN-45 cells (Figure 2C). Meanwhile, similar cellular changes in E-cadherin and Vimentin expression were also certified with IF staining (Figure 2D). Additionally, morphological observation showed that the parental SGC7901 control cells were generally spindle or oval, while SGC7901 RHOJ knockdown cells transformed into the round (Figure S2C), which indicates a trend for GC cells to occur epithelial phenotypic transformation after RHOJ knockdown. As a crucial step in tumor progression, EMT can lose tumor cells' polarity and intercellular adhesion, thereby enhancing the ability of invasion and metastasis. Next, the transwell assay was conducted to evaluate the migration and invasion alterations of GC cells after RHOJ knockdown and RHOJ overexpression. Surprisingly, the migration and invasion ability of GC cells were suppressed by RHOJ knockdown and facilitated by RHOJ overexpression (Figure 2E-F). For cell proliferation standardization of the transwell assay, the CCK-8 assay with a serum-free medium (Figure S2D-E). Given the above, there is a preliminary conclusion that RHOJ mediates EMT to regulate the migration and invasion of GC cells.

### RHOJ promotes the metastasis of GC through EMT *in vivo*

**Figure 3:**
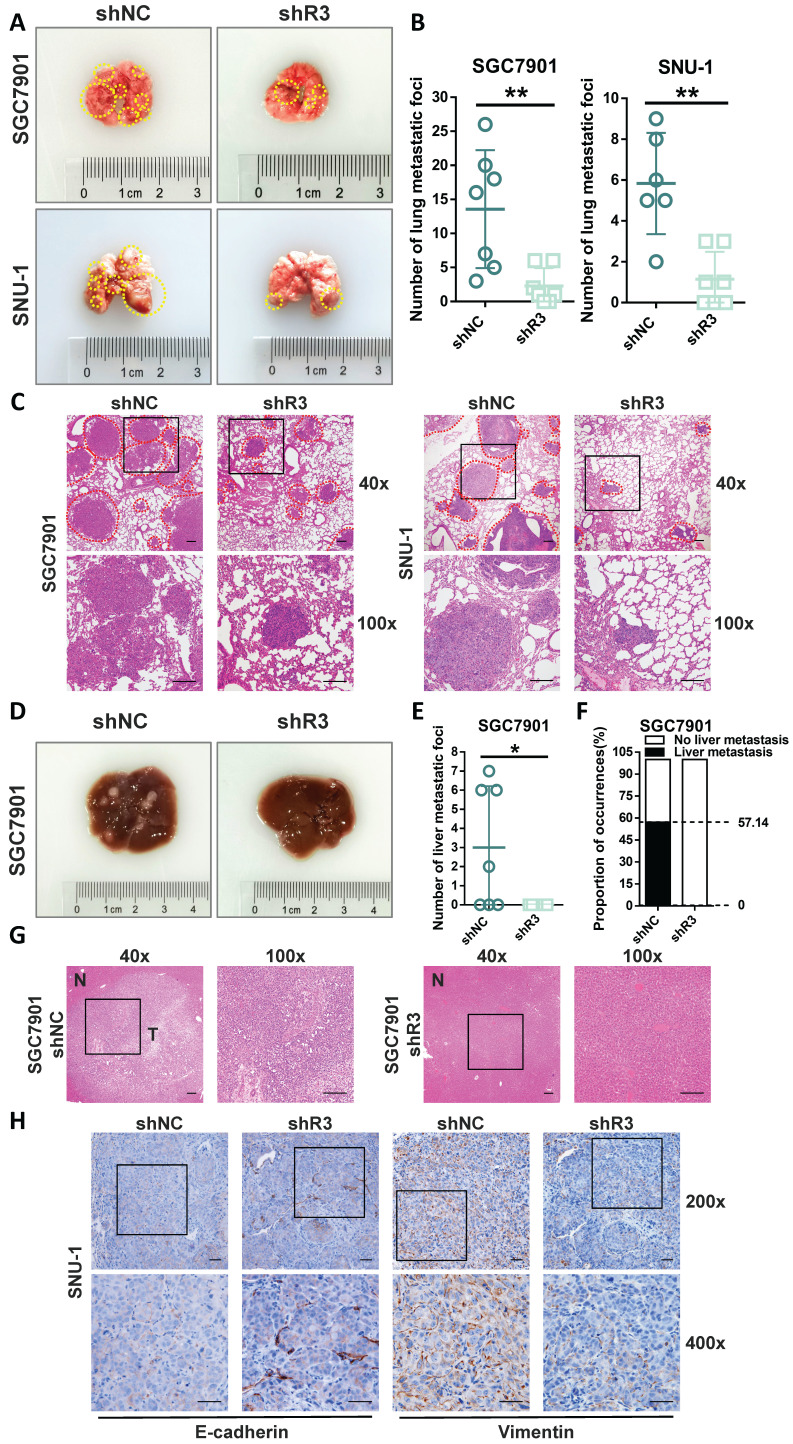
** RHOJ promotes the metastasis of GC through EMT *in vivo*.** (A) Representative photographs of mice lung metastatic foci (SGC7901, SNU-1) in the tail vein metastasis model. (B) Statistics on the number of mice lung surface metastatic foci from (A). (C) Representative images of HE staining from (A), scale bar, 200 µm. (D) Representative photographs of mice liver metastatic foci (SGC7901) in the tail vein metastasis model. (E) Statistics on the number of mice liver surface metastatic foci from (D). (F) Mice liver metastasis rate of GC cells (SGC7901) in the tail vein metastasis model. (G) Representative images of HE staining from (D), tumor tissues and normal tissues were noted as T and N, respectively, scale bar, 200 µm. (H) IHC staining representative images of E-cadherin and Vimentin in mice lung metastatic foci, scale bar, 50 µm. *P<0.05, **P<0.01, ***P<0.001, ****P<0.0001. Data were expressed as Mean±SD.

Next, the tail vein metastasis model of NCG mice was used to examine the effect of RHOJ on GC metastasis *in vivo*. As is shown in Figure 3A-B, the volume and number of metastatic lung foci decreased noticeably by RHOJ knockdown, and similar results were verified by HE staining between the control group and the RHOJ knockdown group as well (Figure 3C). Additionally, the metastatic foci differences in the liver between the two groups were the same as in the lung. As is shown in Figure 3D-G, the RHOJ knockdown group appeared the fewer liver metastatic foci and a lower proportion (57.14% vs 0%) of liver metastasis compared to the control group. Moreover, we performed IHC staining with the sections of lung metastatic foci and showed that the protein levels of E-cadherin and Vimentin were upregulated and downregulated separately in RHOJ knockdown group tumor tissues (Figure 3H), which were in line with the results that described above experiments *in vitro*. Thus, these data indicate that RHOJ promotes the metastasis of GC through EMT *in vivo*.

### RHOJ enhances the angiogenesis of GC

**Figure 4:**
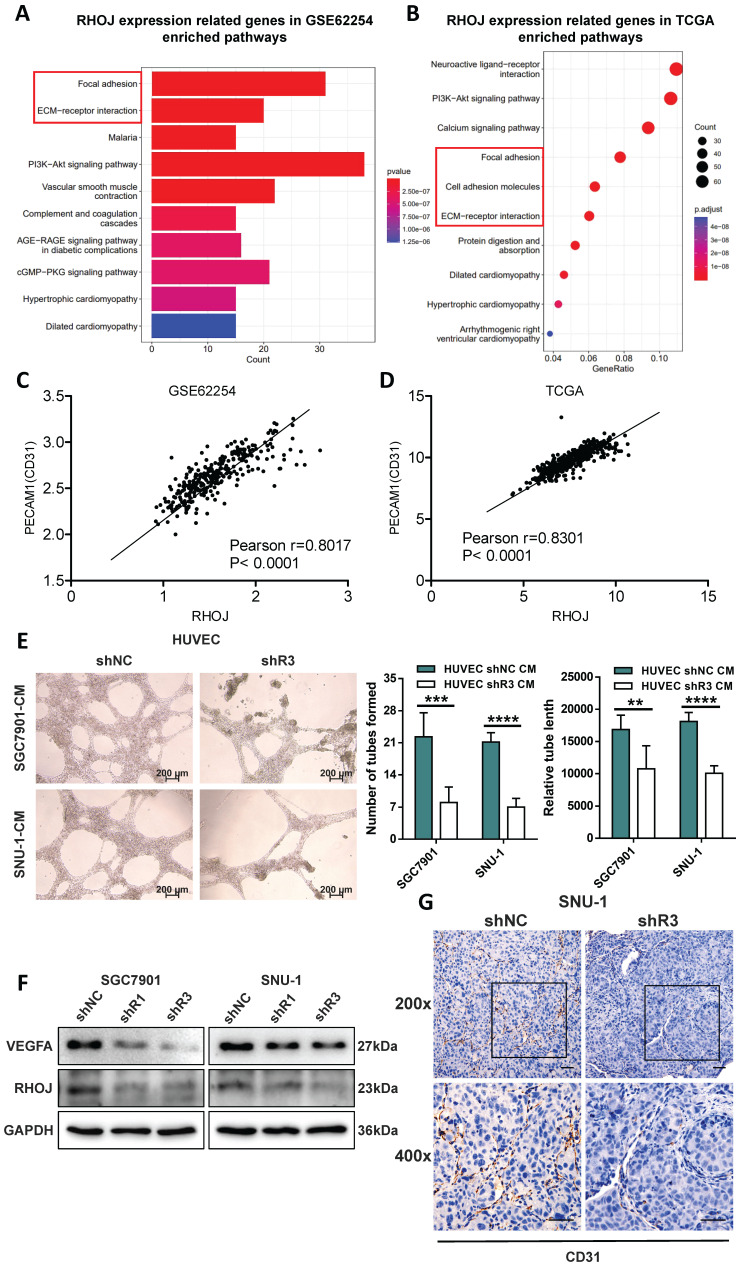
** RHOJ enhances the angiogenesis of GC.** (A) Pathway enrichment analysis of the genes highly related to RHOJ expression, based on the GSE62254 dataset. (B) Pathway enrichment analysis of the genes highly related to RHOJ expression, based on the TCGA database. (C) Pearson correlation analysis evaluated the links between the expression levels of PECAM1 (CD31) with RHOJ in the GSE62254 dataset. (D) Pearson correlation analysis evaluated the links between the expression levels of PECAM1 (CD31) with RHOJ in the TCGA database. (E) Representative images and statistical charts of the formed tubes in HUVEC angiogenesis assay, scale bar, 200 μm. (F) Protein levels of RHOJ and VEGFA were detected by western blotting in RHOJ knockdown cells (SGC7901, SNU-1). (G) IHC staining representative images of CD31 in mice lung metastatic foci, scale bar, 50 μm. *P<0.05, **P<0.01, ***P<0.001, ****P<0.0001. Data were expressed as Mean±SD.

Initiating during tumor progression, angiogenesis brings rich nutrients and oxygen, removes metabolites and carbon dioxide for tumor growth, and provides convenient access for tumor metastasis. Considering the significance of RHOJ in ECs motility and vascular morphogenesis, we next measured the effect of RHOJ on GC angiogenesis *in vitro* and *in vivo*. Firstly, based on the GSE62254 dataset and the TCGA database, the genes which closely related to RHOJ expression were recruited and enriched into the signaling pathway or biological function. As is shown in Figure 4A-B, the focal adhesion, cell adhesion molecules, and extracellular matrix receptor (ECM-receptor) interaction had a close correlation with downstream RHOJ. All of these biological functions were actively involved in tumor metastasis and angiogenesis. As a demonstration of ECs and an indicator for evaluating tumor angiogenesis, CD31 is also known as PECAM1. Pearson correlation analysis displayed a high linear correlation between the expression level of CD31 with RHOJ both in the GSE62254 dataset (Figure 4C) and the TCGA database (Figure 4D). Next, the angiogenesis assay demonstrated impaired tubular structures, fewer tube-formed numbers, and shorter tube lengths by HUVEC in the RHOJ knockdown-conditioned medium group (Figure 4E). Concurrently, western blotting results revealed that RHOJ knockdown in SGC7901 and SNU-1 cells significantly decreased the protein level of VEGFA, which is viewed as a key regulator of angiogenesis during the growth of solid tumors (Figure 4F). In addition, blood vessels in tumor tissues were labeled with CD31 in the sections of metastatic lung foci from NCG mice, and the results confirmed that the number and density of angiogenesis in the RHOJ knockdown group were lower than that in the control group (Figure 4G). Collectively, these data dedicate that RHOJ enhances the angiogenesis of GC.

### RHOJ facilitates GC cells' proliferation and tumor growth

**Figure 5:**
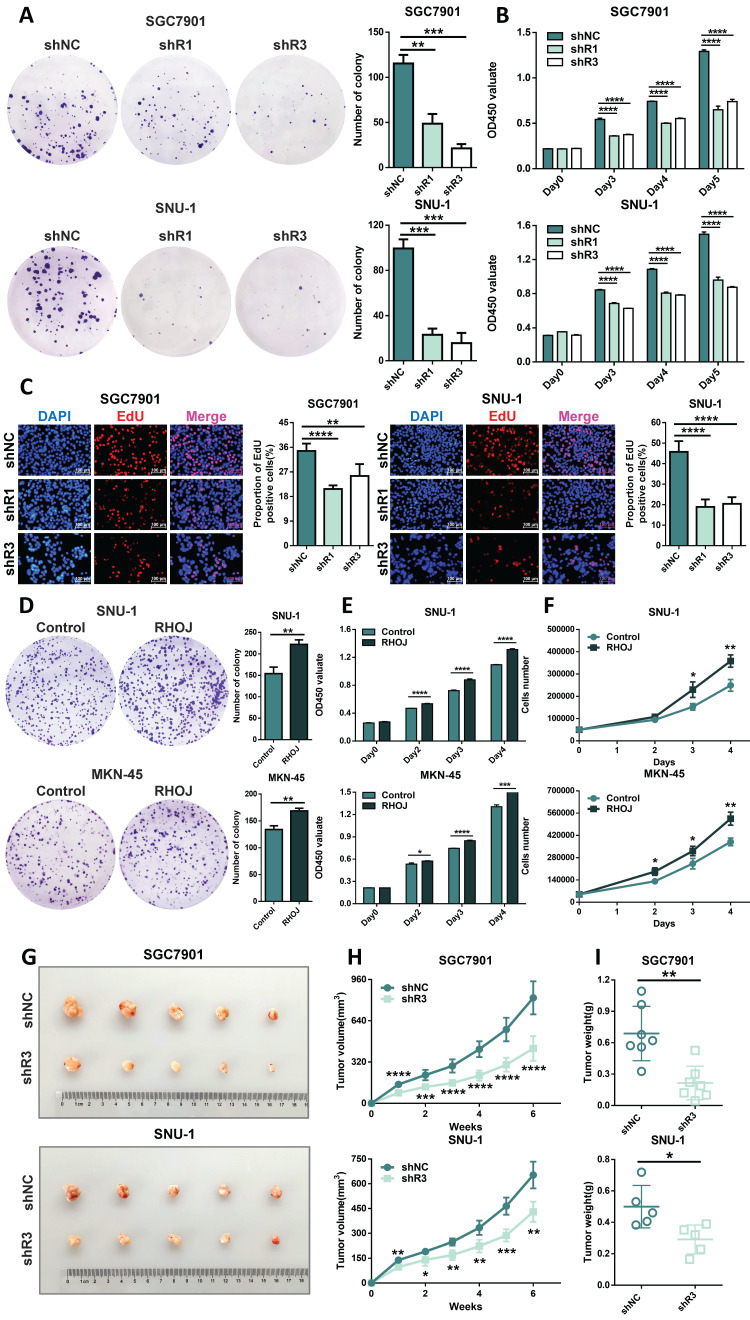
** RHOJ facilitates GC cells**'** proliferation and tumor growth.** (A) Colony-forming assay measured the proliferation ability of RHOJ knockdown cells (SGC7901, SNU-1). (B) CCK-8 assay assessed the viability of RHOJ knockdown cells (SGC7901, SNU-1) at 0 h, 72 h, 96 h, and 120 h, respectively. (C) Representative images and statistical charts of EdU proliferation assay in RHOJ knockdown cells (SGC7901, SNU-1), scale bar, 100 µm. (D) Colony-forming assay measured the proliferation ability of RHOJ overexpression cells (SNU-1, MKN-45). (E) CCK-8 assay assessed the viability of RHOJ overexpression cells (SNU-1, MKN-45) at 0 h, 48 h, 72 h, and 96 h, respectively. (F) Cell counting assay recorded the growth of RHOJ overexpression cells (SNU-1, MKN-45). (G) Representative photographs of mice back tumors (SGC7901, SNU-1) in the subcutaneous xenograft model. (H) The volumes of mice back tumors were recorded weekly with Vernier calipers. (I) Tumors were weighed and counted in each group from (G). *P<0.05, **P<0.01, ***P<0.001, ****P<0.0001. Data were expressed as Mean±SD.

Except for metastasis, uncontrolled proliferation is also a major feature of tumors, and we also tested whether RHOJ can regulate the proliferation of GC cells. As is illustrated in Figure 5A-B, RHOJ knockdown suppressed the proliferation of GC cells (SGC7901, SNU-1). And EdU proliferation assay also indicated that the percentage of EdU-positive cells was decreased after RHOJ knockdown (Figure 5C). Conversely, RHOJ overexpression promoted the proliferation of GC cells (SNU-1, MKN-45) was observed *in vitro* in Figure 5D-F. Likewise, *in vivo*, compared to the control group, the subcutaneous xenograft model of NCG mice showed smaller tumor volume, slower growth rate, and lighter tumor weight in the RHOJ knockdown group (SGC7901, SNU-1) (Figure 5G-I). Taken together, these findings indicate that RHOJ can facilitate GC cells' proliferation and tumor growth as well.

### RHOJ induces S and G2/M phase transition and inhibits GC cells' apoptosis

**Figure 6:**
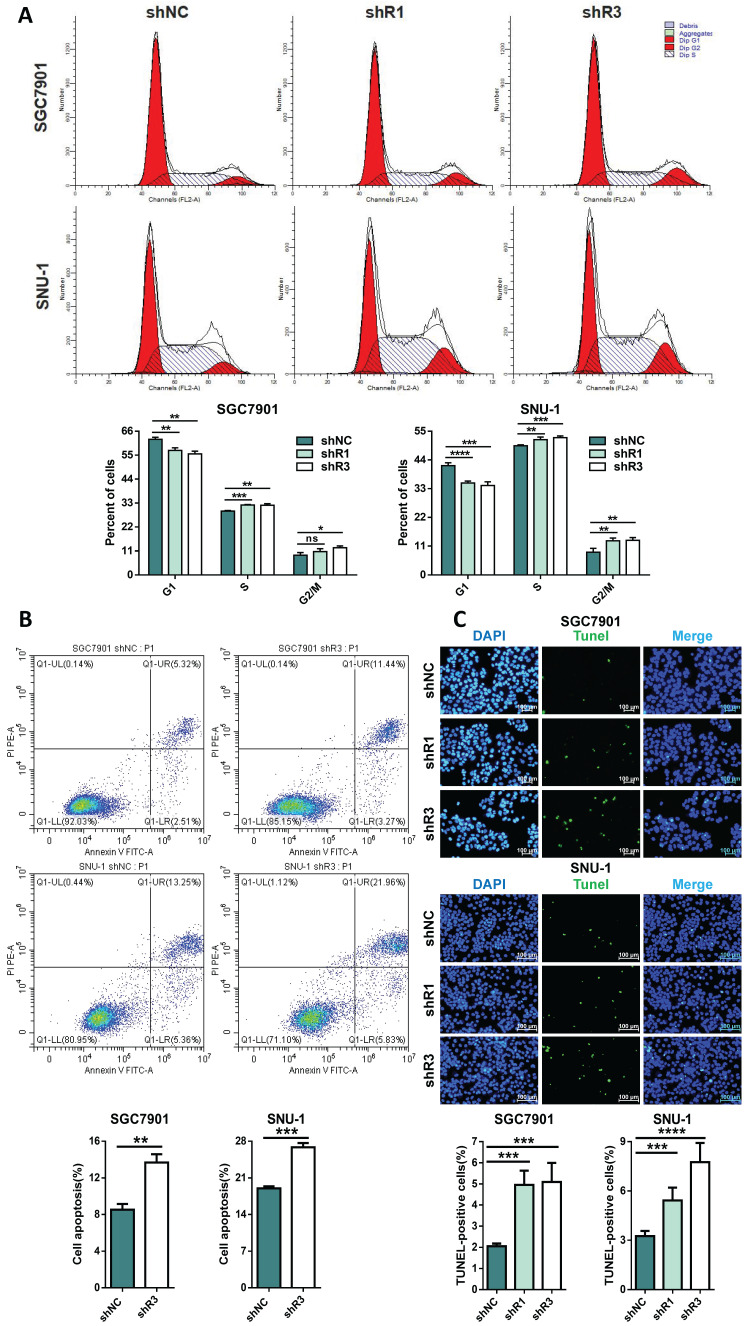
** RHOJ induces S and G2/M phase transition and inhibits GC cells**'** apoptosis.** (A) Flow cytometry analyzed the cell cycles of RHOJ knockdown cells (SGC7901 and SNU-1) and displayed the percentage in the G1, S, and G2/M phases of each group cell with statistical charts. (B) Annexin V-FITC/PI staining assay analyzed the percentage of apoptosis in RHOJ knockdown cells (SGC7901 and SNU-1) and displayed it with statistical charts. (C) Representative images and statistical charts of TUNEL assay in RHOJ knockdown cells (SGC7901, SNU-1), scale bar, 100 µm. *P<0.05, **P<0.01, ***P<0.001, ****P<0.0001. Data were expressed as Mean±SD.

Figure 5 showed a promotive effect of RHOJ on GC cells' proliferation. Rapid division and resistance to apoptosis are common reasons for the infinite proliferation of most tumors, and then the influences of RHOJ on the GC cells' cycle and apoptosis were further investigated. For one thing, flow cytometer analysis suggested that RHOJ knockdown decreased the percentage of GC cells (SGC7901, SNU-1) in the G1 phase and led to cell-cycle arrest in the S phase and G2/M phase (Figure 6A); for another thing, RHOJ knockdown has also been verified to induce cells apoptosis. As is shown in Figure 6B, RHOJ knockdown enhanced GC cells' apoptosis rate by 6%-10% (SGC7901, SNU-1), according to the Annexin V-FITC/PI staining assay. And TUNEL assay also intuitively indicated that the percentage of TUNEL-positive cells increased dramatically after RHOJ knockdown (Figure 6C). In a word, these above results suggest that RHOJ induces S and G2/M phase transition and inhibits cell apoptosis in GC.

### RHOJ regulates the EMT of GC via IL-6/STAT3 signaling

**Figure 7:**
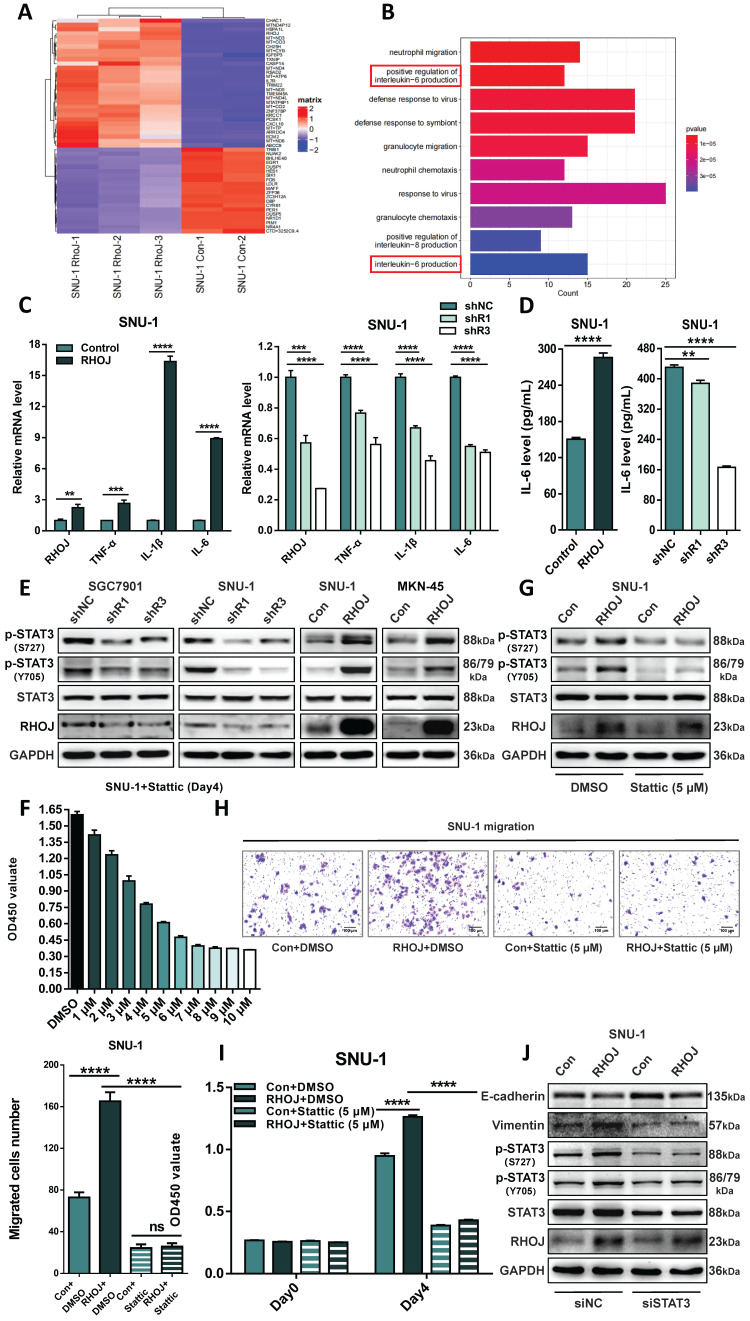
** RHOJ regulates the EMT of GC via IL-6/STAT3 signaling.** (A) Heatmap of the top 50 differentially expressed genes in RHOJ overexpression cells (SNU-1) identified by RNA-seq. (B) GO enrichment analysis of the upregulated genes in RNA-seq data from (A). (C) Relative expression levels of RHOJ, TNF-α, IL-1β, and IL-6 were assessed by qPCR in RHOJ overexpression cells (SNU-1) and RHOJ knockdown cells (SNU-1). (D) IL-6 levels were tested by ELISA in RHOJ overexpression cells (SNU-1) and RHOJ knockdown cells (SNU-1). (E) Protein levels of RHOJ, STAT3, p-STAT3 (Y705), and p-STAT3 (S727) were detected by western blotting in RHOJ knockdown cells (SGC7901, SNU-1) and RHOJ overexpression cells (SNU-1, MKN-45). (F) Treated with different concentrations of Stattic for 96 h, the CCK-8 assay assessed the viability of SNU-1 cells. (G) RHOJ overexpression and control cells (SNU-1) were treated with or without Stattic (5 µM) for 1.5 h, protein levels of RHOJ, STAT3, p-STAT3 (Y705), and p-STAT3 (S727) were detected by western blotting. (H) Representative images and statistical charts of transwell migration assay in RHOJ overexpression and control cells (SNU-1) that were treated with or without Stattic (5 µM), scale bar, 100 µm. (I) Treated with or without Stattic (5 µM) for 96 h, the CCK-8 assay assessed the viability of RHOJ overexpression and control cells (SNU-1). (J) RHOJ overexpression and control cells (SNU-1) were transfected with siRHOJ, protein levels of RHOJ, STAT3, p-STAT3 (Y705), p-STAT3 (S727), Vimentin, and E-cadherin were detected by western blotting. *P<0.05, **P<0.01, ***P<0.001, ****P<0.0001. Data were expressed as Mean±SD.

As of now, the molecular basis that RHOJ regulates the EMT of GC remains unclear. To solve that, RHOJ overexpression and control SNU-1 cells were analyzed by RNA-seq, and the top 50 differentially expressed genes that were closely related to RHOJ expression were recruited by GO enrichment (Figure 7A). Figure 7B listed the top 10 enriched biological processes and suggested that RHOJ may regulate the EMT of GC by influencing interleukin-6 (IL-6) production. Then, the effect of RHOJ on genes' expression in IL-6 signaling (TNF-α, IL-1β, and IL-6) was detected by qPCR.

In SNU-1 cells, the expression levels of TNF-α, IL-1β, and IL-6 were upregulated with RHOJ overexpression and were downregulated with RHOJ knockdown respectively (Figure 7C). Meanwhile, similar changes in IL-6 levels between RHOJ overexpression and RHOJ knockdown cells (SNU-1) were also certified with ELISA (Figure 7D). As a classical downstream molecule of IL-6, STAT3 has been reported to exert an important regulatory role in the EMT of tumors 30-32. We detected the expression of crucial proteins (STAT3, p-STAT3 (Y705), and p-STAT3 (S727)) within IL-6/STAT3 signaling via western blotting. As is displayed in Figure 7E, p-STAT3 (Y705) and p-STAT3 (S727) were tremendously downgraded in SGC7901 and SNU-1 cells after RHOJ knockdown, while in SNU-1 and MKN-45 cells, RHOJ overexpression showed a reversed alteration. To further confirm the necessity of IL-6/STAT3 signaling for RHOJ to regulate EMT in GC cells, Stattic (HY-13818, MCE, Shanghai, China), a specific chemical inhibitor of STAT3, was used to block the IL-6/STAT3 signaling. CCK-8 assay found that Stattic was able to inhibit GC cells' proliferation with a concentration-dependent phenomenon, and a rational concentration (5 µM) was selected for further experiments (Figure 7F). Treated with Stattic in RHOJ overexpression and control SNU-1 cells, RHOJ and STAT3 itself remained static, while p-STAT3 (Y705) and p-STAT3 (S727) were interrupted obviously, according to western blotting (Figure 7G). Next, the Transwell assay observed RHOJ overexpression enhanced the migrative ability of SNU-1 cells, while RHOJ-mediated migration was blocked by Stattic (Figure 7H). Similarly, treatment with Stattic also significantly reversed the promotive effect of RHOJ on the proliferation ability of SNU-1 cells (Figure 7I). Additionally, incubated with siSTAT3 in SNU-1 cells, the expression of STAT3, p-STAT3 (Y705), p-STAT3 (S727), and Vimentin were subsequently downregulated, E-cadherin is upregulated, while RHOJ remained constant (Figure 7J). This indicated that after STAT3 suppression, its phosphorylation level is also downregulated, and the EMT process by RHOJ-inducing was weakened, while as an upstream molecule of those, RHOJ, remains static. Therefore, these data suggest that the activation of phosphorylation STAT3 in IL-6/STAT3 signaling was necessary for RHOJ to regulate the EMT in GC cells.

## Discussion

Even though slow declines in incidence have been observed in many countries worldwide for decades, GC remains one of the most terrible cancers of the world (especially in Asia) due to its highly aggressive and metastatic ability, and anti-chemotherapy effect. Therefore, the difficulties and highlights of many studies about GC focus on overcoming its metastasis and drug resistance. Diagnosis, screening, and individual treatment of GC patients are benefited from the deep exploration of GC molecular pathology in the past decades. Where the ACRG subtypes of GC not only promote the prognosis prediction for patients in clinical work but also provide lots of creative guidance for studies and treatments of GC.

In this work, based on the GSE62254 dataset firstly, the EMT-subtype GC owning the shortest OS and DFS was observed in which RHOJ was upregulated significantly compared with the other three GC subtypes of ACRG and had a close relationship with the EMT of GC. Subsequently, the hypothesis that RHOJ induces the EMT to promote the invasion and migration of GC cells was certified by a series of assays *in vitro and in vivo*. In many reports about Rho GTPases in cell biology, the CDC42 subfamily (CDC42, TC10, TCL/RHOJ, Chp, and Wrch-1) was studied extensively in the past decades. CDC42 contracts and polymerizes actin through the Arp2/3 complex, and induces co-linking and remodeling to form a cytoskeleton; CDC42 in active status facilitates microtubule terminal binding proteins via enhancing the combination between CLIP170 and IQGAP1 (an effector of CDC42 and RAC), in turn, affects cytoskeleton and cell polarity 33, 34. Characterized by tumor cells having the strongest motility and metastasis ability, the EMT-subtype GC patients have the poorest prognosis routinely. Cell migration is a dynamic process driven by the cytoskeleton, and the transformation of epithelial cells into mesenchymal-like morphology in EMT is closely related to cytoskeletal alterations as well. Our previous studies found RHOJ aggravates the progression and invasion of glioblastoma by impairing cytoskeleton dynamics 28. All these findings further verify the vital role of RHOJ in poor prognosis of the EMT-subtype GC and suggest cytoskeletal alterations may be also partially involved in the regulated effects of RHOJ on the EMT and migration of GC cells.

Tumor cells invade blood vessels and spread into the bloodstream is the critical pathway of distant metastasis of malignancy, the quantity and density of blood vessels in tumor tissues are the key factors of influence tumor cells to spread into the blood. Therefore, weakened GC angiogenesis was observed after RHOJ knockdown by the angiogenesis assay of HUVEC and IHC staining of CD31 in this study. Previous studies showed the multifaceted role of RHO GTPases in ECs morphogenesis and angiogenesis, including ECs migration and capillaries established 35. Regulated by the transcription factor ERG, RHOJ is an endothelial cell-restricted RHO GTPase that mediates vascular morphogenesis by promoting the lumen formation of ECs 15. Combined with our study, there is a hypothesis that targeting RHOJ may improve the poor prognosis of GC (especially EMT-subtype GC) patients. In the past decades, compounds or antibody drugs targetting RHO GTPases are one of the key directions in new drug development, and researchers found that the drugs that block RHO GTPases signaling can inhibit tumor growth and angiogenesis 36. Using the APT_EDB_-LS complex (a highly selected drug carrier for tumor tissues), Sunghyun Kim et al. effectively delivered siRHOJ into tumor tissues and established a way to clinically inhibit RHOJ by the *in vivo* siRNA delivery system 37. Further studies from Chan Kim et al. certified that RHOJ blockade is an effective and selective strategy for tumor angiogenesis and vascular disruption, and also showed that it has the advantages of good curative effect, high specificity, and mild toxic side effects 27. Nevertheless, so far, there are still no specific small molecule inhibitors or antibody drugs targeting RHOJ for follow-up efficacy studies and safety evaluations, which is also an urgent problem for us to focus on next.

About the molecular basis, RNA-seq and using a specific inhibitor of STAT3 unveiled RHOJ activates STAT3 phosphorylation through IL-6/STAT3 signaling to induce EMT in GC. Generally, as a pleiotropic cytokine, IL-6 plays a central role in human physiological and pathophysiological responses, such as participating in inflammation, autoimmune diseases, and cancer. Inducing the EMT process and angiogenesis, the IL-6/STAT3 signaling acts an important effect in many solid tumor progressions, such as hepatocellular carcinoma and colorectal cancer 38-42. Although it was verified that RHOJ can upregulate IL-6 expression, the question of how RHOJ activates the production of IL-6 is still being explored further. Some reported that NF-κB activation is a key course involved in the process of RHO GTPases to regulate IL-6 and IL-8 expression 43, 44. However, the question of the mechanism that stimulates NF-κB activation is still ambiguous. In vascular smooth muscle cells, induced by vasoconstrictor angiotensin II, RHOA mediates phospho-Ser536 nuclear factor κB/RelA subunit exchange on the IL-6 promoter, thereby mediates IL-6 expression and vascular inflammation 45. RHO GTPases (RHOA, RAC1, and CDC42) are novel signal transducers for substance P-stimulated IL-8 expression in human colonic epithelial cells 46. Overall, existing reports are suggested that the activation of NF-κB and IL-6 promoters may be a potential channel in the process of RHOJ promoting the production of IL-6, the subsequent studies will be deeply verified by mass spectrometry analysis, co-immunoprecipitation, and luciferase reporter assay, etc.

NF-κB and IL-6/STAT3 signalings are important regulators in tumor inflammation. The crosstalks between its and RHO GTPases prove RHO GTPases are involved in tumor inflammation and microenvironment. Activation of proinflammatory signalings promotes tumor cell proliferation, survival, chemoresistance, angiogenesis, and immune cell infiltration. Here, according to our findings that the upregulation of mRNA levels of TNF-α, IL-1β, and IL-6 after RHOJ overexpression, it further indicates the potential proinflammatory effect of RHOJ in GC. Except for the above critical factors, RHO GTPases also contribute to tumor cells to occur immune escape 47. Not only inhibits tumor growth by enhancing phagocytosis to tumor cells, but RHO-associated kinase (ROCK) block also initiates T cells and anti-tumor immunity, where the proportion of CD8+ T cells increases significantly 48. Contrastly, ROCK-myosin II activation in melanoma cells can differentiate to CD163+ CD206+ protumorigenic macrophages by monocyte chemotaxis. ROCK blocking reduced Treg cell infiltration *in vivo* and improved efficacy when ROCK inhibitor was combined with immunotherapy in murine melanomas. The following problems remain to be further studied whether RHOJ-mediated signaling devotes to developing anti-tumor immunity in GC cells and whether the combination of small molecule compounds targeting RHOJ and immunosuppressants can improve the prognosis of GC patients.

## Conclusions

**Figure 8:**
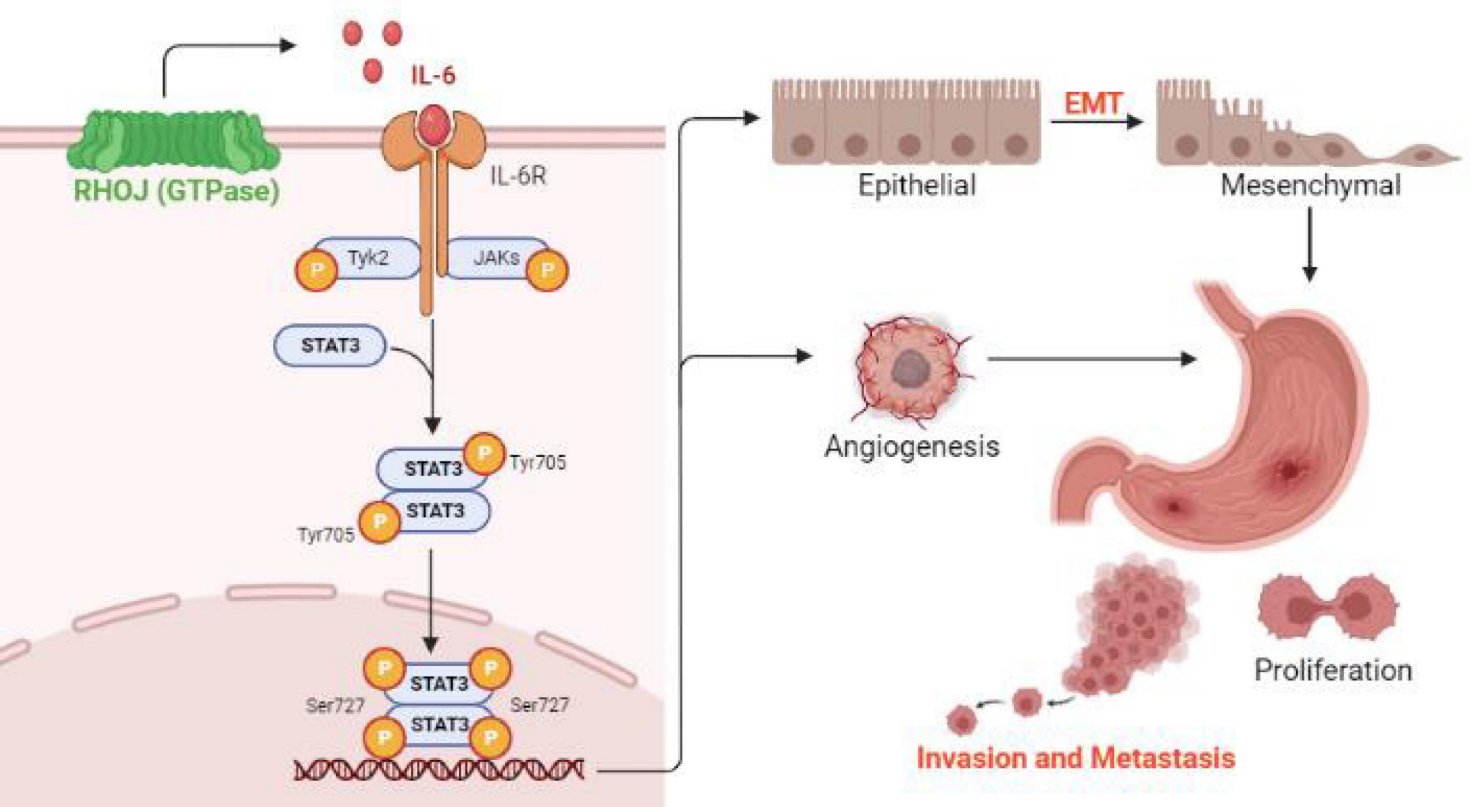
** Schematic illustration of RHOJ induces EMT by IL-6/STAT3 to promote invasion and metastasis in GC.** RHOJ activates the IL-6 production firstly and enhances the phosphorylation level of STAT3 via IL-6/STAT3 signaling, which induces the EMT process of GC cells and angiogenesis, further promoting GC invasion, metastasis, and tumor growth.

Based on the findings RHOJ is upregulated notably in EMT-subtype GC and associated with patients' poor survival, after RHOJ overexpression observed that RHOJ contributes to GC cells' EMT, migration, invasion, tumor growth, and metastasis. Blocking the IL-6/STAT3 pathway overcomes RHOJ-mediated GC cells' migration and growth. Thus, this study reveals a novel metastatic mechanism of GC that RHOJ induces EMT by IL-6/STAT3 to promote invasion and metastasis in GC (Figure 8), and RHOJ is identified as a new oncogene that induces GC metastasis and may become a promising prognostic marker and therapeutic target for EMT-subtype GC patients.

## Supplementary Material

Supplementary materials and methods, figures and tables.
