# Supplementary material for: RHOJ Induces Epithelial-to-Mesenchymal Transition by IL-6/STAT3 to Promote Invasion and Metastasis in Gastric Cancer
Source: Int J Biol Sci. 2023 Aug 21;19(14):4411–26. doi: 10.7150/ijbs.81972 (PMC10535698; doi:10.7150/ijbs.81972)
Supplement: Supplementary file 1 — Supplementary materials and methods, figures and tables. [file ijbsv19p4411s1.pdf]

RHOJ Induces Epithelial-Mesenchymal Transition by IL-6/STAT3 to Promote Invasion and Metastasis in Gastric Cancer

Supplementary materials and methods

Tissue specimens

A total of 30 formalin-fixed, paraffin-embedded GC samples (8 EMT-subtype GC samples and 22 Non-EMT-subtype-GC samples) were randomly recruited from the pathology department of Nanjing Drum Tower Hospital. All of these samples were cut into 4 μm slices and used for IHC staining.

Cell culture

Human GC cells line (MKN-45, MKN-28, MGC-803, BGC-823, SNU-1, HGC-27, SGC7901) and human umbilical vein endothelial cells (HUVEC) were purchased from iCell Bioscience Inc (Shanghai, China). Human GC cells line (SNU-719, SNU-216) and human embryonic kidney 293T (HEK-293T) cells were obtained from were derived from Cobioer Biosciences (Nanjing, China). Maintained at 37°C and 5% CO_2, all cells were cultured in DMEM medium (Gibco, USA) supplemented with 10% fetal bovine serum (Sigma, USA) and 1% penicillin/streptomycin (Sangon Biotech, Shanghai, China).

Establishment of stable RHOJ knockdown/overexpression GC cells line

To generate the stable RHOJ knockdown/overexpression GC cell line, lentiviral constructs of RHOJ knockdown and RHOJ overexpression were used. pLKO.1-shScramble and pLKO.1-shRHOJ vectors were acquired from

Sigma-Aldrich. pLenti-CMV-GFP/puro and pLenti-CMV-RHOJ vectors were purchased from Miaoling Biotechnology (Wuhan, China). pLKO.1-shRHOJ vector sequences were as follows: pLKO.1-shRHOJ-1 (forward) 5'-GCCCGTTTGCTGTATATGAAA-3', pLKO.1-shRHOJ-3 (forward) 5'-CATCTGCTTCTCTGTCGTAAA-3'. Briefly, pLKO.1-shRHOJ plasmids containing the RHOJ knockdown target sequences were transduced into SGC7901 and SNU-1 cells, while pLenti-CMV-RHOJ plasmids containing the RHOJ overexpression target sequences were transduced into SNU-1 and MKN-45 cells. After 48 h of infection with corresponding lentiviral constructs, puromycin-resistant single clones were screened (screening concentration was 2 μg/mL). Approximately after 4 weeks, the single clones proliferated and the RHOJ knockdown/overexpression clones were identified by qPCR and western blotting.

Quantitative real-time PCR (qPCR)

Total RNA was extracted from GC cells using the Total RNA rapid extraction reagent (Yfxbio, Nanjing, China). qPCR was performed using a 5×All-In-One RT MasterMix (G592, ABMgood, USA) and Hieff® qPCR SYBR Green Master Mix (11201ES50, Yeasen, Shanghai, China), according to the manufacturer's instruction. The following primer sequences were selected: GAPDH, (forward) 5'-ACCCAGAAGACTGTGGATGG-3', (reverse) 5'-TTCAGCTCAGGGATGACCTT-3', RHOJ, (forward) 5'-CCTGAGTGACAGAGAAAGAACC-3', (reverse) 5'-GGAGTGTGTGCGTATGAAAGA-3', TNF-α, (forward)

5'-GAGGCCAAGCCCTGGTATG-3', (reverse) 5'-CGGGCCGATTGATCTCAGC-3', IL-1β, (forward) 5'-TGAAATGATGGCTTATTACAGTGG-3', (reverse) 5'-GTAGTGGTGGTCGGAGATTCGTAG-3', IL-6, (forward) 5'-GGCACTGGCAGAAAACAACC-3', (reverse) 5'-GCAAGTCTCCTCATTGAATCC-3'.

Western blotting

Total proteins from GC cells were homogenized in Lysis Buffer (50 mM Tris, 150 mM NaCl, 2 mM EDTA, 0.5% Nonidet P-40, pH 7.4) containing 1% protease inhibitors and 1% phosphatase inhibitors. After centrifuging at 12,000 rpm for 10 min at 4°C, the total protein content was measured by Pierce BCA Protein Assay Kit (23227, Thermo) and denatured for 10 min at 100°C. Proteins were separated by 10% SDS-PAGE and transferred to nitrocellulose membranes and blocked for 1.5 h with 5% skim milk (PBS diluted). Then membranes were respectively incubated with the following primary antibodies: RHOJ (1:500, Abnova, H00057381-M01), E-cadherin (1:1000, Cell Signaling Technology, #3195), Vimentin (1:1000, Cell Signaling Technology, #5741), N-cadherin (1:2000, Proteintech, 22018-1-AP), ZEB1 (1:1000, Abclonal, A5600), SNAI2 (1:1000, Proteintech, 12129-1-AP), STAT3 (1:5000, Thermo, MA1-13042), p-STAT3 (Tyr705) (1:2000, Cell Signaling Technology, #9145S), p-STAT3-S727 (1:1000, Abclonal, AP0715), VEGFA (1:1100, Sangon, D360788-0025), and GAPDH (1:100000, Proteintech, 60004-1-Ig), and then were detected using specific secondary antibodies (1:10000, Bioworlde, BS13278/BS12478). Protein bands were visualized by the ECL kit (P10200, NCM

Biotech, Suzhou, China). The relative levels of all individual proteins were based on GAPDH.

Immunofluorescence (IF)

Seeded in co-focal dishes (2×10^5 cells/well), cells were fixed with 4% paraformaldehyde and then ruptured the cell membranes by 0.3% Triton (PBS diluted) for 10 min. After three times of PBS washing, non-specific antigen-binding sites were blocked for 1.5 h with 5% BSA (PBS diluted). Cells were then separately incubated with E-cadherin (1:100, Abclonal, A11492) and Vimentin (1:100, Proteintech, 60330-1-Ig) overnight at 4°C. Next day, after three times of PBS washing, cells were incubated with corresponding secondary antibodies attached with FITC (1:100, Abclonal, AS001) or Cy3 (1:100, Abclonal, AS007) for 1.5 h. The nuclei were then stained with DAPI for 5 min and terminal images were captured using a confocal microscope from the Analysis and Testing Center at NMU.

Immunohistochemistry (IHC)

Human GC samples and mice lungs samples were fixed with 4% paraformaldehyde and paraffin-embedded before cutting it into 4 μm thick sections. The tissue sections were routinely dewaxed, rehydrated, and subjected to antigen retrieval by heating in sodium citrate (pH 6.0) for 10 min. After blocking with 3% H_2O_2, sections were incubated with the primary antibody (5% BSA diluted): RHOJ (1:150, ORIGENE, TA505592), E-cadherin (1:400, Cell Signaling Technology, #3195), Vimentin (1:400, Cell Signaling Technology, #5741), and CD31 (1:400, Cell Signaling Technology, #77699) at 4°C for overnight, next day washed with PBS and incubated with the

corresponding secondary antibody (PV-9001/PV-9002, ZSGB-BIO, Beijing, China) for 30 min at 37°C. DAB kit (PV-9001/PV-9002, ZSGB-BIO, Beijing, China) was applied to visualize the sections, followed by counterstaining with hematoxylin and dehydrating. Terminal images of these sections were captured by a bright-field microscope (Leica Microsystems).

Collecting conditioned medium

RHOJ knockdown cells (SGC7901, SNU-1) were seeded into 6-well plates (3×10^5 cells/well) and cultured with DMEM complete medium. After 36 h cultivation, sterile syringes and 0.2 μm filters (4433, PALL) were used to collect the cells' supernatant as a conditioned medium. Then its were stored at 4°C and used within a week.

Angiogenesis assay

The conditioned medium of RHOJ knockdown cells (SGC7901, SNU-1) was collected in advance and reserved at 4°C. 1 h prior to running angiogenesis assay, 500 μL diluted matrigel (40183ES08, Yeasen, Shanghai, China) was added to 24-well plates and placed at 37°C for solidification. Then 4×10^5 HUVEC cultured with 500 μL conditioned medium were dripped in 24-well plates. After 16 h incubation, the tube formation images of HUVEC were photographed by a microscope, and the tubes' relative length and number were counted by Image J software.

Colony-forming assay

Cells were plated in 6-well plates (1.5×10^3 cells/well) and cultured for 15 days. Then cells were fixed with 4% paraformaldehyde for 40 min and stained with 0.1% crystal

violet for 40 min. After washing with water, the final figures were photographed and counted by Image J software.

CCK-8 assay

Approximately 2,000 cells per well with 100 μL DMEM complete culture medium were planted in 96-well plates. At 0 h, 48 h, 72 h, 96 h, and 120 h, respectively, 10 μL CCK-8 solution (40203ES76, Yeasen, Shanghai, China) was added to per well. After 1.5 h incubation, the absorbance was measured at 450 nm.

Cell counting assay

Cells were seeded in 12-well plates (5×10^5 cells/well), and each group of cells had three independent wells at least. Then cells were separated from plates using trypsin at 0 h, 48 h, 72 h, and 96 h, respectively, and then blood counting plates were used for cell counting.

Small interfering RNA (siRNA) transfection assay

siRNAs were purchased from Genepharma (Shanghai, China), and the sequence of siSTAT3 was (forward) 5'-GCAGCAGCUGAACAACAUGTT-3'. According to the manufacturer's protocol, siRNAs were transfected into GC cells by siRNA-Mate Transfection Reagent (G04003, Genepharma, Shanghai, China). After 48 h incubation, total proteins were collected for further experiments.

Supplementary figure legends

Figure S1. Upregulated in EMT-subtype GC, RHOJ is correlated with poor GC prognosis, supplemented for Figure 1

(A) In the GSE62254 dataset, RHOJ (labeled by molecular probes 235489_at) expression levels in the four GC subtypes of ACRG.

(B) Kaplan-Meier analysis showed the OS in RHOJ high expression group and low expression group GC patients, according to the TCGA database.

(C) The ingenuity pathway analysis (IPA) of EMT-subtype GC-related genes identified the signaling of RHO Family GTPases.

**P*<0.05, ***P*<0.01, ****P*<0.001, *****P*<0.0001. Data were expressed as Mean± SD.

Figure S2. RHOJ mediates EMT to regulate the migration and invasion of GC cells, supplemented for Figure 2

(A) Pearson correlation analysis assessed the links between the expression levels of the EMT-related genes (CDH1, VIM, ZEB1, ZEB2, and FN1) with RHOJ in the TCGA database.

(B) RHOJ relative expression levels of RHOJ knockdown cells (SGC7901, SNU-1) were assessed by qPCR.

(C) Morphological observation showed morphological variance between parental control and RHOJ knockdown cells (RHOJ), magnification, 100×, 200×, scale bar, 200 μm, 100 μm, respectively.

(D) Cultured with a serum-free medium, the CCK-8 assay assessed the viability of

RHOJ knockdown SGC7901 and SNU-1 cells at 48 h and 24 h, respectively.

(E) Cultured with a serum-free medium, the CCK-8 assay assessed the viability of RHOJ overexpression SNU-1 and MKN-45 cells at 24 h and 36 h, respectively.

*$P<0.05$, **$P<0.01$, ***$P<0.001$, ****$P<0.0001$. Data were expressed as Mean $\pm$ SD.

Figure S1

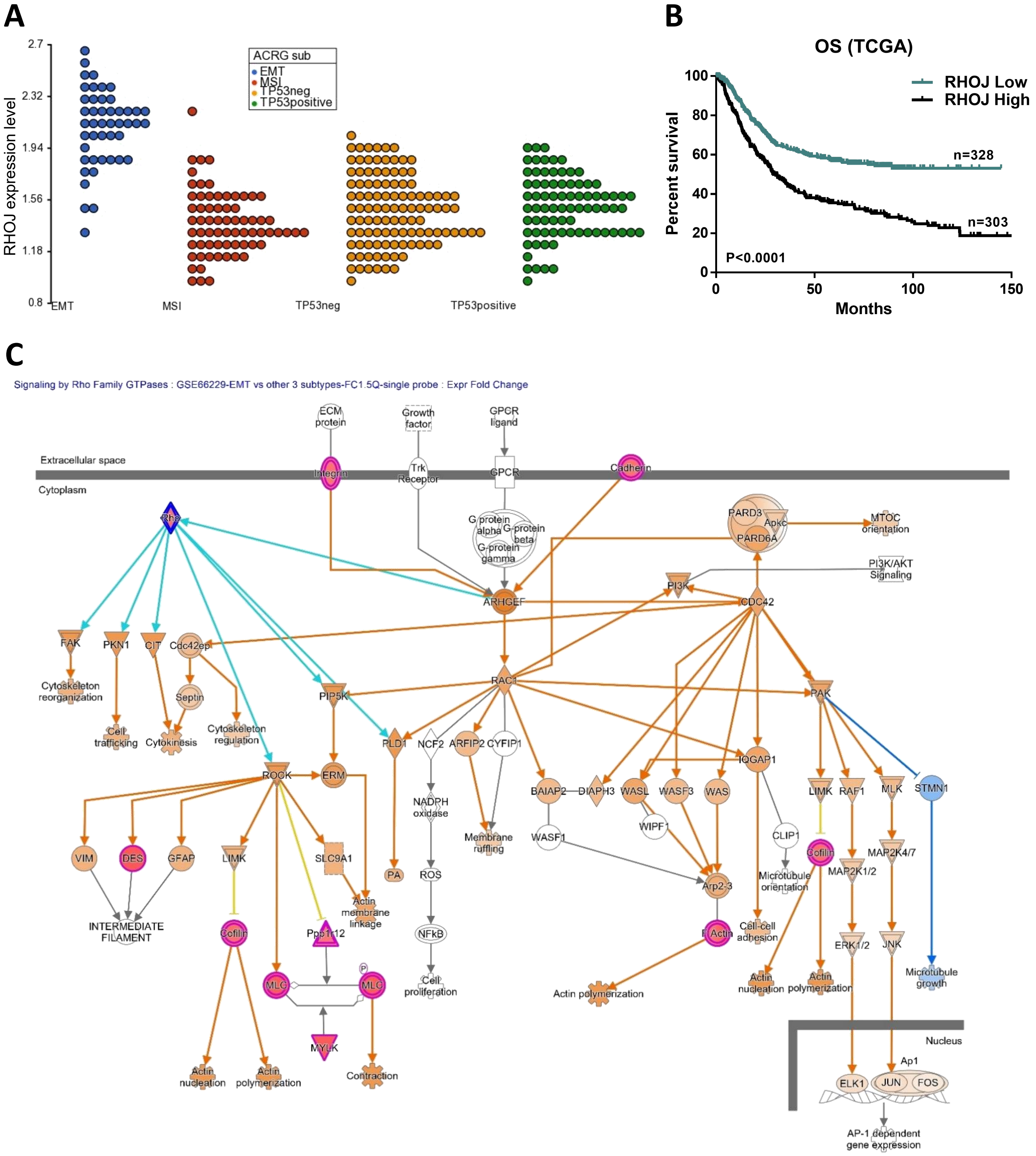
A
RHOJ expression level
2.7
2.32
1.94
1.56
1.18
0.8
ACRG sub
EMT
MSI
TP53neg
TP53positive
EMT
MSI
TP53neg
TP53positive
B
OS (TCGA)
RHOJ Low
RHOJ High
Percent survival
100
80
60
40
20
0
n=328
n=303
P<0.0001
0
50
100
150
Months
C
Signaling by Rho Family GTPases : GSE66229-EMT vs other 3 subtypes-FC1.5Q-single probe : Expr Fold Change
ECM protein
Growth factor
GPCR ligand
Extracellular space
Cytoplasm
Integrin
Trk Receptor
GPCR
Cadherin
Rho
G protein alpha
G-protein beta
G-protein gamma
PARD3
Apkc
PARD6A
MTOC orientation
PI3K/AKT Signaling
PI3K
ARHGEF
CDC42
FAK
PKN1
CIT
Cdc42ep
Cytoskeleton reorganization
Septin
RAC1
PIP5K
PAK
Cell trafficking
Cytokinesis
Cytoskeleton regulation
PLD1
NCF2
ARFIP2
CYFIP1
IQGAP1
ROCK
ERM
BAIAP2
DIAPH3
WASL
WASF3
WAS
LIMK
RAF1
MLK
STMN1
NADPH oxidase
WIPF1
VIM
DES
GFAP
LIMK
SLC9A1
PA
ROS
Membrane ruffling
WASF1
CLIP1
Cofilin
MAP2K4/7
MAP2K1/2
Arp2-3
Microtubule orientation
INTERMEDIATE FILAMENT
Cofilin
Ppp1r12
Actin membrane linkage
NFkB
F Actin
Cell-cell adhesion
ERK1/2
JNK
MLC
MLC
Cell proliferation
Actin polymerization
Actin nucleation
Actin polymerization
Microtubule growth
MYLK
Nucleus
Ap1
Actin nucleation
Actin polymerization
Contraction
ELK1
JUN
FOS
AP-1 dependent gene expression

Figure S2

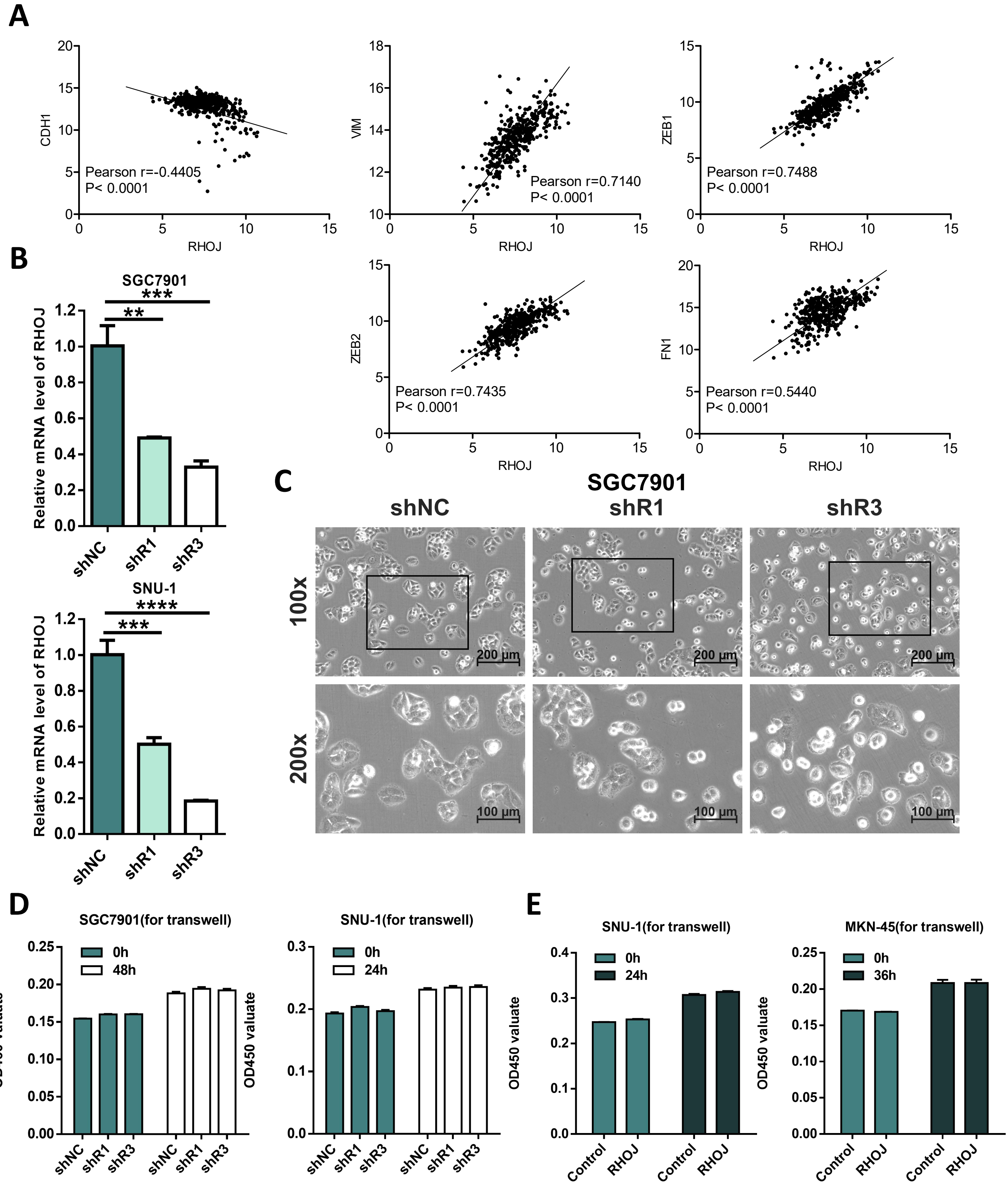

A
CDH1
RHOJ
Pearson r=-0.4405
P< 0.0001
VIM
Pearson r=0.7140
P< 0.0001
ZEB1
Pearson r=0.7488
P< 0.0001
ZEB2
Pearson r=0.7435
P< 0.0001
FN1
Pearson r=0.5440
P< 0.0001
B
SGC7901
SNU-1
Relative mRNA level of RHOJ
shNC
shR1
shR3
C
SGC7901
shNC
shR1
shR3
100x
200x
200 μm
100 μm
D
SGC7901(for transwell)
SNU-1(for transwell)
OD450 valuate
0h
48h
24h
E
SNU-1(for transwell)
MKN-45(for transwell)
36h
Control
RHOJ

Table S1 Correlation between the GC clinicopathological features and expression of RHOJ

Characteristics	Low Expression [N, (%)]	High Expression [N, (%)]	*P*-Value
**Molecular Subtype**			
EMT	3 (2.0)	43 (28.7)	**<0.0001** ****
MSI	47 (31.3)	21 (14.0)	
MSS/TP53-	59 (39.4)	48 (32.0)	
MSS/TP53+	41 (27.3)	38 (25.3)	
**Age**			
Mean (SD)	63.29 (10.64)	60.59 (11.91)	**0.0398** *
Median [Min, Max]	64.0 [31.0, 84.0]	62.0 [24.0, 86.0]	
Not reported	0	0	
**Gender**			
Male	107 (71.3)	92 (61.3)	0.0869
Female	43 (28.7)	58 (38.7)	
**Status**			
Alive	89 (59.3)	59 (39.3)	**0.0008** ***
Dead	61 (40.7)	91 (60.7)	
**Stage (T)**			
T1+T2	117 (78.0)	71 (47.3)	**<0.0001** ****
T3+T4	33 (22.0)	79 (52.7)	
**Stage (N)**			
N0+N1	91 (60.7)	78 (52.0)	0.1623
N2+N3	59 (39.3)	72 (48.0)	
**Stage (M)**			
M0	142 (94.7)	131 (87.3)	**0.0420** *
M1	8 (5.3)	19 (12.7)	
**pStage**			
I	20 (13.3)	12 (8.0)	**0.0029** **
II	60 (40.0)	36 (24.0)	
III	38 (25.4)	57 (38.0)	
IV	32 (21.3)	45 (30.0)	
**Recurrence**			
Yes	46 (30.7)	79 (52.7)	**0.0004** ***
No	95 (63.3)	62 (41.3)	
Unknown	9 (6.0)	9 (6.0)	
**Perineural Invasion**			
Positive	35 (23.3)	53 (35.3)	**0.0364** *
Negative	90 (60.0)	69 (46.0)	
Not reported	25 (16.7)	28 (18.7)	
